# Prognostic Impact of Hypoxia-Inducible *miRNA-210* in Patients with Lung Adenocarcinoma

**DOI:** 10.1155/2015/316745

**Published:** 2015-02-05

**Authors:** Jun Osugi, Yuka Kimura, Yuki Owada, Takuya Inoue, Yuzuru Watanabe, Takumi Yamaura, Mitsuro Fukuhara, Satoshi Muto, Naoyuki Okabe, Yuki Matsumura, Takeo Hasegawa, Athushi Yonechi, Mika Hoshino, Mitsunori Higuchi, Yutaka Shio, Hiroyuki Suzuki, Mitsukazu Gotoh

**Affiliations:** Department of Regenerative Surgery, School of Medicine, Fukushima Medical University, Fukushima 960-1295, Japan

## Abstract

*Objective*. The aim of this study was to investigate the prognostic value of *MicroRNA-210* (*miR-210*) expression in patients with non-small-cell lung cancer (NSCLC). *Methods*. We examined the *miR-210* expression of samples of 80 patients, who underwent surgical resection at Fukushima Medical University from 2004 to 2007, by using quantitative RT-PCR. The relationship between *miR-210* expression and clinicopathological factors as well as histological subtype was statistically analyzed. *Results*. *miR-210* expression showed an inverse correlation with disease-free and overall survival in patients with NSCLC. Significant correlations were found between *miR-210* expression and lymph node metastasis, late disease stages, and poor prognosis in patients with adenocarcinoma. Multivariate Cox analysis indicated that *miR-210* expression was an independent prognostic factor for disease-free survival in patients with adenocarcinoma. *Conclusions*. We showed that *miR-210* may be a prognostic biomarker for patients with NSCLC, especially for those with lung adenocarcinoma.

## 1. Introduction

Hypoxia is a common feature of pathological conditions such as tissue ischemia and inflammation, as well as of the microenvironment of solid tumors [1]. Many cellular responses to hypoxia are thought to be mediated through changes in targeted gene expression. One major mechanism mediating cellular responses to hypoxia is the pathway of hypoxia inducible factor-1 (HIF-1) [2]. HIF-1 is a member of the basic helix-loop-helix/Per-Arnt-Sim (bHLH-PAS) family of proteins and binds to hypoxia-response elements (HRE) in the promoters of target genes. HIF-1 consists of an alpha (HIF-1*α*) and a beta (HIF-1*β*) subunit and activates the expression of at least 150 genes, which encode proteins that regulate cell metabolism, cell cycle, proliferation, apoptosis, autophagy, erythropoiesis, immune reactions, cytokine production, and angiogenesis as well as many other functions [3]. HIF-1*β* is a non-oxygen-responsive nuclear protein. In contrast, HIF-1*α* is highly inducible by hypoxia [4].

In human cancers, HIF-1*α* is overexpressed as a result of intratumoral hypoxia and of genetic alterations affecting crucial oncogenes and tumor suppressor genes [3]. HIF-1*α* overexpression has been associated with increased patient mortality in many different human cancers [3]. Similarly, HIF-1*α* overexpression has been reported at both the protein [5, 6] and the mRNA [7, 8] level in non-small-cell lung cancer (NSCLC) patients with poor prognosis. In preclinical studies, inhibition of HIF-1*α* activity has marked effects on tumor growth; inhibitors of HIF-1*α* have therefore attracted much attention as new therapeutic agents for patients with advanced malignancies, and several clinical studies have been performed [3]. Research has shown that HIF-1*α* antagonists, such as EZN-2968 and PX-478, inhibit tumor cell proliferation* in vitro* and* in vivo* [9, 10].

miRNAs have emerged as a new class of noncoding genes that are involved in the regulation of cell proliferation, differentiation, and viability [11]. miRNAs are single-stranded small RNA molecules of approximately 22 nucleotides that silence the expression of target genes either through mRNA degradation or suppression of transcription [12–14]. The miRNAs that are regulated by hypoxia were examined in a 2007 study in which* miR-210* was identified as the most consistently and robustly induced miRNA in hypoxic cells and tissues [15].* miR-210* expression is frequently elevated in a variety of cancers [15], including lung cancer [16–20].* miR-210* is regulated by both HIF-1*α* [21–23] and HIF-2*α* [24], and a recent study demonstrated that HIF-1*α* directly binds to an HRE on the proximal* miR-210* promoter [23].* miR-210* plays numerous crucial roles in the cellular response to hypoxia, such as in apoptosis [15, 25], angiogenesis [26], cell cycle regulation [24, 27], DNA damage repair [22], mitochondrial metabolism [28, 29], and tumor growth [19]. Furthermore,* miR-210* is also involved in stem cell biology [30]. Thus,* miR-210* is thought to have essential roles in tumorigenesis along with HIF-1*α*.

It has been reported that* miR-210* overexpression is correlated with poor prognosis in breast [21, 31], pancreatic [32], and head and neck cancer patients [31]. Recently, two systematic reviews and a meta-analysis confirmed that* miR-210* is useful for prediction of the survival of patients with various tumors, especially breast cancers [33, 34]. However, these two studies did not include the outcome of patients with lung cancer. Therefore, the prognostic impact of* miR-210* in patients with lung cancer remains unclear. Within this context, we analyzed* miR-210* expression in NSCLC patient samples, and showed that it could be a prognostic biomarker, especially for patients with adenocarcinoma.

## 2. Materials and Methods

### 2.1. Patient and Tissue Samples

In total, 80 snap-frozen NSCLC and 30 matched normal adjacent lung tissue samples were evaluated for* miR-210* expression. These consecutive samples were obtained from patients who underwent surgical resection at the Department of Regenerative Surgery, Fukushima Medical University, Fukushima, Japan, from January 2004 to December 2007. The clinical characteristics of the 80 patients included in this study were typical of the characteristics of resected NSCLC reported by the Japan Lung Cancer Society (2004) with respect to age, sex, histology, and pathological stage [35]. None of the patients had received any previous anticancer treatment. Ethical approval for analysis of samples and patient notes was obtained from the local research ethics committee. Tumor types and stages were determined according to the 7th edition of Union for International Cancer Control TNM classification. At the time of surgery, all tissue samples were immediately frozen in liquid nitrogen and stored at −80°C until assay. All samples were analyzed histologically to assess the amount of tumor component (at least 70% tumor cells) and the quality of the material (i.e., absence of necrosis). These 80 cases consisted of 34 female and 46 male patients with a median age of 69 years (range: 51–85), of which 54 were stage I cases, 12 were stage II, and 14 were stage III. The median observation period was 74.5 months (range: 5–117), and the five-year survival rate was 62.4%. Full patient clinicopathological characteristics are provided in Table 1.

### 2.2. RNA Extraction and Quantitative Real-Time PCR

TaqMan-based quantitative real-time polymerase chain reaction (qRT-PCR) was applied to assess miRNA expression levels in tissue samples. RNA was extracted from snap-frozen lung tumor samples or normal lung tissue using the miRVana miRNA Isolation Kit (Ambion, Austin TX, USA). The quantity and quality of the extracted RNA were determined spectrophotometrically by measurement of absorbance at 260 and 280 nm using a DU530 UV-VIS spectrophotometer (Beckman Coulter, Fullerton, CA, USA). Samples with a 260/280 ratio of 1.80 or greater were used for analysis. miRNA-cDNA was synthesized from 5 ng of total RNA using microRNA-specific primers and the TaqMan MicroRNA Reverse Transcription kit (Applied Biosystems, Foster City, CA, USA). The TaqMan MicroRNA assays for* miR-210* (ID: 000512) and* RNU6B* (ID: 001093) were purchased from Applied Biosystems. Real-time PCR was performed in triplicate using a StepOnePlus Real-Time PCR system (Applied Biosystems). For miRNA assays, each PCR reaction contained 1.33 *μ*L reverse transcription product, 2×TaqMan Universal Master Mix, and 1 *μ*L TaqMan MicroRNA assay. The 20 *μ*L reactions were incubated in a 96-well optical plate at 95°C for 10 minutes, followed by 40 cycles at 95°C for 15 seconds, and 60°C for 60 seconds. Changes in miRNA expression between treatment and controls were determined using the 2^−ΔΔCt^ method [36], and results were normalized against* RNU6B* expression levels. For lung cancer samples, the controls consisted of the median of 30 normal lung tissues.

### 2.3. Statistical Analysis

Correlations between the status of* miR-210* expression and clinical characteristics were assessed using Student's *t*-test, Pearson and Spearman's rank test, or the Mann-Whitney *U* test. Kaplan-Meier survival analysis was performed by applying the long-rank test to* miR-210* expression and was stratified by median values and quartiles. Disease-specific overall survival (referred to as overall survival hereafter) was defined as the time from surgery to last follow-up or time of NSCLC-specific death. Disease-free survival was defined as the time from surgery to the time of first evidence of radiographic metastatic disease. Univariate and multivariate analyses were performed using the Cox proportional hazard model. All statistical analyses were performed using SPSS 17 software (SPSS Inc., Chicago, IL, USA), and *P* values of <0.05 were considered significant.

## 3. Results

### 3.1. Relationship between* miR-210* Expression and Clinical Characteristics in Patients with NSCLC

To examine whether miR-210 expression correlates with clinical characteristics in patients with NSCLC, we analyzed* miR-210* expression of NSCLC samples using qRT-PCR. For each sample, the data were normalized using* RNU6B* as a reference. The fold-change in* miR-210* expression for each NSCLC sample was calculated by comparison with the median of 30 normal control samples. Patients were divided into two subgroups according to high or low* miR-210* expression levels. Patients with higher than the median expression level of* miR-210* were defined as the high group of* miR-210* expression. No significant association between the status of* miR-210* expression and clinical characteristics such as sex, age, tumor size, histology, *T* factor, lymph node status, pathological stage, ly factor, or *v* factor was observed. However, we found that the status of* miR-210* expression was significantly correlated with disease relapse (*P* = 0.007, Pearson and Spearman's rank test; Table 1).

### 3.2. *miR-210* Expression Is a Prognostic Factor for Disease-Free Survival and Overall Survival of NSCLC Patients

**Figure 1:**
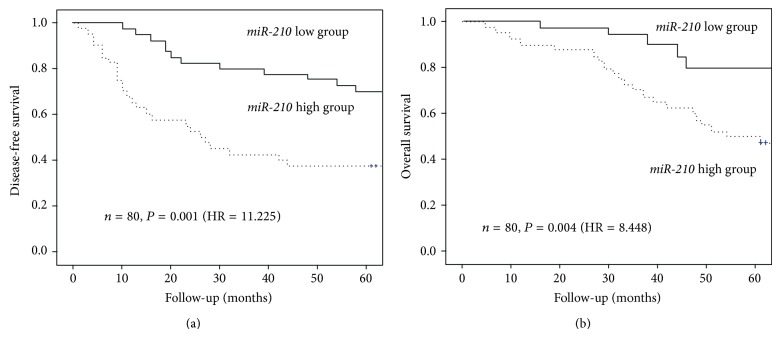
Kaplan-Meier curves of survival of patients with NSCLC (*n* = 80). Kaplan-Meier curves of (a) disease-free and (b) overall survival of 80 patients with NSCLC, stratified according to* miR-210* levels. Expression levels were stratified by the median value; follow-up was limited to 60 months.

To confirm the correlation between* miR-210* and prognosis, we analyzed the relationship between* miR-210* expression and patient survival by performing Kaplan-Meier survival analysis, applying the long-rank test to* miR-210* expression. The* miR-210*-high group showed significantly shorter disease-free survival than the* miR-210*-low group (log rank chi-square = 11.225, *P* = 0.001; Figure 1(a)). Five-year disease-free survival was 37.5% in the* miR-210*-high group and 70.0% in the* miR-210*-low group. Furthermore, the* miR-210*-high group showed significantly shorter overall survival than the* miR-210*-low group. Five-year overall survival was 47.5% in the* miR-210*-high group and 77.5% in the* miR-210*-low group (log rank chi-square = 8.448,* P* = 0.004; Figure 1(b)).

### 3.3. *miR-210* Expression Is Significantly Associated with Important Clinicopathological Factors in Patients with Lung Adenocarcinoma

Although we found that* miR-210* expression is a prognostic factor for disease-free survival and overall survival in NSCLC patient samples; the status of* miR-210* expression was not significantly correlated with important clinicopathological factors. We therefore analyzed the correlation of each histological type with* miR-210* expression and clinical characteristics. In 62 patient samples with adenocarcinoma,* miR-210* expression was significantly correlated with lymph node status, pathological stage, ly factor, *v* factor, and disease relapse (*P* = 0.018, *P* = 0.003, *P* = 0.0009, *P* = 0.044, and *P* = 0.002, resp., Mann-Whitney *U* test), whereas, in 18 patient samples with squamous cell carcinoma, it was not significantly correlated with any clinical characteristic (Table 2).

### 3.4. *miR-210* Expression Is a Prognostic Factor for Disease-Free and Overall Survival in Patients with Lung Adenocarcinoma

**Figure 2:**
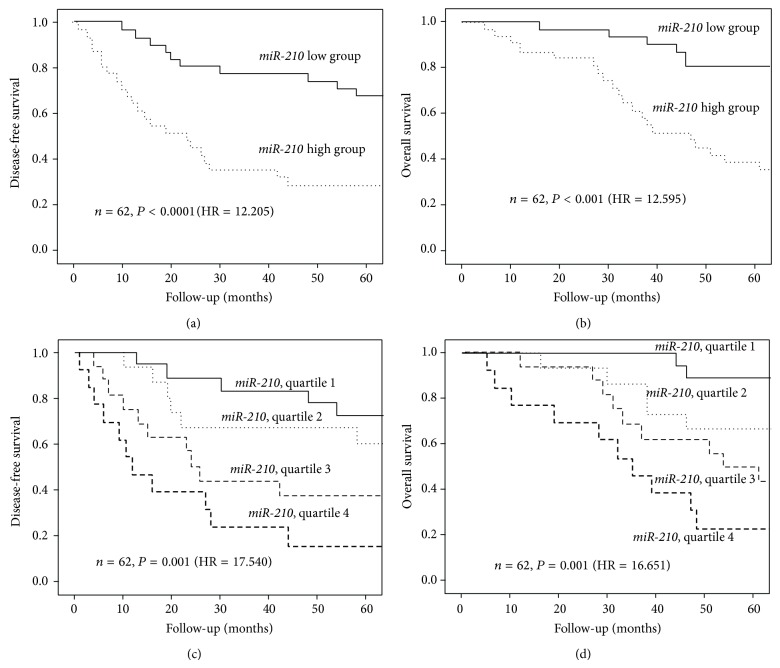
Kaplan-Meier survival curves for patients with adenocarcinoma (*n* = 62). Kaplan-Meier curves were constructed of the following: (a) disease-free and (b) overall survival of 62 patients with adenocarcinoma stratified according to* miR-210* levels. Expression levels were stratified by the median value; follow-up was limited to 60 months. (c) Disease-free and (d) overall survival of patients with adenocarcinoma stratified according to* miR-210* levels. Expression levels were stratified by quartiles; follow-up was limited to 60 months.

Because* miR-210* expression in adenocarcinoma patient samples was correlated with many important clinicopathological factors, we hypothesized that* miR-210* expression would be more closely correlated with prognosis in adenocarcinoma patient samples than in NSCLC patient samples. To test this hypothesis, Kaplan-Meier survival analysis was performed by applying the log-rank test to* miR-210* expression of 62 patient samples with adenocarcinoma.* miR-210* expression was a strong adverse prognostic factor for disease-free and overall survival when considered as a binary variable divided by median value (log rank chi-square = 12.205, *P* < 0.001; Figure 2(a), log rank chi-square = 12.595, *P* < 0.001; Figure 2(b), resp.). However, in the 18 patient samples with squamous cell carcinoma, no significant correlation between* miR-210* expression and disease-free or overall survival was observed. In the 62 lung adenocarcinoma patient samples, these relationships were also statistically significant when the patients were divided into quartiles on the basis of miR-210 expression levels; miR-210 expression was an adverse prognostic factor for disease-free and overall survival (log rank chi-square = 17.540, *P* < 0.001; Figure 2(c), log rank chi-square = 16.651, *P* = 0.001; Figure 2(d), resp.).

### 3.5. Multivariate Analysis Indicated That* miR-210* Expression in Adenocarcinoma Patient Samples Is an Independent Prognostic Factor for Disease-Free Survival

We performed further studies of diagnostic factors of disease-free survival and overall survival, specifically in adenocarcinoma patients, using univariate and multivariate analysis. These data are summarized in Table 3. In a Cox univariate analysis, sex, tumor size, *T* factor, lymph node metastases, p-stage, ly factor, *v* factor, and* miR-210* expression were significantly correlated with disease-free survival (*P* = 0.024, *P* = 0.034, *P* = 0.036, *P* < 0.0001, *P* < 0.0001, *P* = 0.001, *P* = 0.004, and *P* = 0.001, resp.), whereas only age was not correlated with disease-free survival. Furthermore, a Cox multivariate analysis defined sex, ly factor, and* miR-210* expression as independent prognostic factors for disease-free survival (*P* = 0.008, *P* = 0.021, and *P* = 0.020, resp.). Moreover, in a Cox univariate analysis, sex, tumor size, *T* factor, lymph node metastases, p-stage, ly factor, *v* factor, and* miR-210* expression were also significantly correlated with overall survival (*P* = 0.005, *P* = 0.044, *P* = 0.018, *P* = 0.001, *P* < 0.0001, *P* = 0.004, *P* = 0.032, and *P* = 0.001, resp.). Furthermore, Cox multivariate analysis showed that the usefulness of* miR-210* as a prognostic factor for overall survival was marginal (*P* = 0.057).

## 4. Discussion

By univariate and multivariate analyses our study provides clear evidence that upregulation of* miR-210* is a prognostic factor in patients with lung adenocarcinoma and is correlated with important clinicopathological factors including nodal involvement, pathological stage, lymphatic vessel invasion, and cancer relapse.

Recently, a meta-analysis of human lung cancer microRNA expression profiling studies that compared cancer tissues with normal tissues showed that the top two most consistently reported upregulated microRNAs were* miR-210* and* miR-21* [37]. In addition, systematic reviews and meta-analyses of two studies confirmed that upregulation of* miR-210* is predictive of poor survival of patients with various tumors, especially breast cancers [33, 34]. However, these two systematic reviews did not include the outcome of patients with lung cancer. Thus, the prognostic impact of* miR-210* in patients with lung cancer remained unclear.

Recently, Eilertsen et al. reported a large-scale study of the prognostic role of* miR-210* in NSCLC [38]. In that study, upregulation of* miR-210* expression was a positive prognostic factor for disease-free survival in 335 NSCLC patients. This result is not consistent with our findings. One reason for the differences between these results might be the different methods used, since our study assessed* miR-210* expression using qRT-PCR, whereas the previous study used* in situ* hybridization. However, other previous studies strongly suggest that high expression of* miR-210* could be a biomarker of bad prognosis in lung cancer. Puisségur et al. reported that* miR-210* was significantly elevated in patients with advanced disease such as stages II-III disease compared with stage I A disease (*n* = 20) [39]. Li et al. reported that miR-210 was significantly elevated in patients with stages III-IV disease compared with stages I-II disease (*n* = 60) [40]. Furthermore, in the majority of the previous studies,* miR-210* upregulation was significantly correlated with poor prognosis in patients with various cancers such as breast cancer [21], pancreatic cancer [32], head and neck cancer [31], colorectal cancer [41], and glioblastoma [42], though not renal cancer [43]. Our data are consistent with these previous important findings. Further study is still needed to clarify the clinical impact of* miR-210* as a prognostic factor in patients with NSCLC.

*In vitro* functional studies regarding* miR-210* function in cancer progression provide even further contradictory results. For example, Zhang et al. found that* miR-210* inhibits MNT, an antagonist of c-MYC, and promotes cell proliferation in transformed cells such as colon and cervical cancer cells [24]. However, Giannakakis et al. found that* miR-210* acts as a tumor suppressor by inhibiting cell proliferation via E2F3 regulation in ovarian cancer cell lines [27]. It is unclear how to reconcile these paradoxical findings that* miR-210* acts primarily as a positive or a negative regulator of proliferation.

To understand these conflicting findings, we hypothesized that* miR-210* may play various roles depending on the cancer type or histological subtype in which it is expressed. In the present study, we first analyzed the correlation of* miR-210* expression in NSCLC patient samples with each histological subtype of NSCLC and with the clinical characteristics of patients with each subtype. We then focused on* miR-210* expression in samples with histology specific for adenocarcinoma. In patients with adenocarcinoma, we clearly showed that* miR-210* expression was strongly associated with important clinical parameters such as age, lymph node metastasis, pathological stage, ly factor, *v* factor, and relapse, while, in patients with squamous cell carcinoma,* miR-210* was not associated with any clinical characteristic. In our study, squamous cell carcinoma showed high levels of baseline* miR-210* expression compared with adenocarcinoma. The uniformly high expression levels of* miR-210* in most squamous cell carcinomas meant that a prognostic impact of* miR-210* on squamous cell carcinoma could not be determined. However,* miR-210* could be a biomarker of adenocarcinoma because adenocarcinomas showed varying levels of* miR-210* expression.

## 5. Conclusions

In conclusion, this study demonstrated for the first time that* miR-210* was correlated with poor prognosis in patients with NSCLC, especially in lung adenocarcinoma. This evidence could contribute to biomarker studies in patients with lung adenocarcinoma. Because this was an exploratory study and the sample size was very small, our results warrant further investigation and require independent validation. In particular,* miR-210* levels in the plasma may have special prognostic significance since miRNA has been shown to circulate in various body fluids in remarkably stable forms, resulting in the identification of novel noninvasive biomarkers for the diagnosis and prognosis of various cancers and other diseases [44, 45]. Based on these evidences, the prognostic signature of* miR-210* in the plasma of NSCLC patients should be examined in future studies.

## Figures and Tables

**Table 1:** Relationship between *miR-210* expression and clinicopathological factors.

Clinicopathological factor	Patient number	*miR-210* miRNA expression		*P*
		High group	Low group	
Gender				0.258
Male	46	26	20	
Female	34	14	20	
Age				0.222
≤65	24	9	15	
≥66	56	31	25	
Tumor size				0.82
<30 mm	48	23	25	
≥30 mm	32	17	15	
T factor				0.481
T1-T2	61	34	37	
T3-T4	9	6	3	
Lymph node				0.293
Negative	61	28	33	
Positive	19	12	7	
P-stage				0.232
Stage I	54	24	30	
Stages II-III	26	16	10	
ly factor				0.502
Negative	41	19	22	
Positive	39	21	17	
v factor				0.359
Negative	43	22	27	
Positive	27	18	13	
Relapse				0.007^*^
With	37	25	12	
Without	43	15	28	

Relapse was defined as first evidence of radiographic metastatic disease after surgery.

^*^ *P* < 0.05.

**Table 2:** Relationship between *miR-210* expression and clinical characteristics.

Clinical characteristics	*miR-210* expression			
	Ad cases	*P*	Sq cases	*P*
Gender		0.086		1.000
Male	2.32 ± 2.89		2.88 ± 2.00	
Female	1.45 ± 1.67		2.93 ± 0.88	
Age		0.029^*^		—
≤65	1.18 ± 1.71		—	
≥66	2.17 ± 2.60		2.87 ± 1.89	
Tumor size		0.402		1.000
<30 mm	1.84 ± 1.95		2.87 ± 1.62	
≥30 mm	1.87 ± 2.98		3.25 ± 2.14	
Histology		—		—
Adeno	1.86 ± 2.40		—	
Squamous	—		2.87 ± 1.89	
T factor		0.417		1.000
T1-T2	1.82 ± 2.48		2.88 ± 1.65	
T3-T4	2.34 ± 1.70		3.37 ± 4.40	
Lymph node		0.034^*^		—
Negative	1.44 ± 2.01		2.87 ± 1.89	
Positive	2.34 ± 2.97		—	
P-stage		0.019^*^		1.000
Stage I	1.37 ± 2.02		2.88 ± 1.65	
Stages II-III	2.62 ± 2.77		3.37 ± 4.40	
ly factor		0.025^*^		0.620
Negative	1.23 ± 1.44		3.30 ± 2.07	
Positive	2.24 ± 2.84		2.09 ± 1.23	
v factor		0.097		1.000
Negative	1.75 ± 1.97		2.12 ± 1.84	
Positive	2.34 ± 2.98		3.01 ± 1.86	
Relapse		0.007^*^		1.000
With	2.72 ± 2.63		2.88 ± 1.96	
Without	1.35 ± 1.75		2.87 ± 1.84	

Ad: adenocarcinoma.

Sq: squamous cell carcinoma.

Relapse was defined as first evidence of radiographic metastatic disease after surgery.

^*^ *P* < 0.05.

**Table:** (a) Disease-free survival

Variables		Univariate analysis		Multivariate analysis	
		HR (95% CI)	*P*	HR (95% CI)	*P*
Gender	Male/female	2.263 (1.113–4.601)	0.024^*^	3.600 (1.392–9.309)	0.008^*^
Age	≥66/≤65	0.860 (0.427–1.730)	0.672		
Tumor size	≥33/<33	2.130 (1.052–4.735)	0.034^*^	2.310 (0.819–6.518)	0.114
T factor	T1/T2–T4	2.232 (1.052–4.735)	0.036^*^	0.469 (0.134–1.645)	0.237
Lymph node	+/−	3.754 (1.857–7.590)	<0.0001^*^	1.038 (0.300–3.589)	0.953
P-stage	I/II-III	4.969 (2.396–10.304)	<0.0001^*^	3.270 (0.894–11.964)	0.073
ly factor	+/−	4.078 (1.822–9.128)	0.001^*^	4.027 (1.229–13.193)	0.021^*^
v factor	+/−	2.768 (1.376–5.566)	0.004^*^	0.994 (0.412–2.398)	0.989
*miR-210 *	High/Low	0.284 (0.134–0.604)	0.001^*^	0.355 (0.148–0.847)	0.02^*^

**Table:** (b) Overall survival

Variables		Univariate analysis		Multivariate analysis	
		HR (95% CI)	*P*	HR (95% CI)	*P*
Gender	Male/female	3.218 (1.434–7.222)	0.005^*^	2.151 (1.889–14.047)	0.001^*^
Age	≥66/≤65	1.011 (0.473–2.162)	0.977		
Tumor size	≥33/<33	2.173 (1.019–4.630)	0.044^*^	1.725 (0.618–4.811)	0.298
T factor	T1/T2–T4	2.988 (1.204–7.415)	0.018^*^	0.801 (0.211–3.039)	0.744
Lymph node	+/−	3.453 (1.614–7.391)	0.001^*^	1.858 (0.499–6.918)	0.356
P-stage	I/II-III	4.533 (2.027–10.138)	<0.0001^*^	1.492 (0.380–5.853)	0.567
ly factor	+/−	3.865 (1.554–9.615)	0.004^*^	3.387 (0.894–12.836)	0.073
v factor	+/−	2.303 (1.075–4.937)	0.032^*^	0.602 (0.226–1.607)	0.311
*miR-210 *	High/low	0.235 (0.099–0.561)	0.001^*^	0.361 (0.127–1.031)	0.057

CI: confidence interval.

^*^ *P* < 0.05.
